# Single shot adductor canal block combined with intravenous patient-controlled analgesia can be effective as continuous adductor canal block in reducing opioid consumption and breakthrough pain after total knee arthroplasty

**DOI:** 10.1186/s40634-022-00523-6

**Published:** 2022-08-23

**Authors:** Sung Eun Kim, Hyuk-Soo Han, Myung Chul Lee, Du Hyun Ro

**Affiliations:** 1 Department of Orthopedic Surgery, Seoul National University College of Medicine, Seoul, South Korea; 2 Department of Orthopedic Surgery, Seoul National University Hospital, 101 Daehak-ro, Jongno-gu, Seoul, South Korea

**Keywords:** Adductor canal block, Intravenous patient-controlled analgesia, Pain management, Total knee arthroplasty

## Abstract

**Purpose:**

The aim of this study was to compare the following three analgesic methods after Total knee arthroplasty (TKA): intravenous patient-controlled analgesia (IV-PCA), continuous adductor canal block (C-ACB), and intravenous patient-controlled analgesia combined with single shot adductor canal block (PCA + sACB).

**Methods:**

Records of 482 patients undergoing primary TKA from September 2019 to September 2020 were analyzed. Patients were divided into three pain control groups: IV-PCA (*n* = 180), C-ACB (*n* = 173) and PCA + sACB (*n* = 129). Single shot adductor canal block was performed 24 h after surgery in the PCA + sACB group. Rescue opioid consumption, breakthrough pain, pain numerical rating scale (NRS), and anti-emetics administration were measured from postoperative day (POD) 1 to POD 5.

**Results:**

Rescue opioid consumption was less in C-ACB or PCA + sACB group than in the IV-PCA group at POD1 (*p* < 0.001 and *p* = 0.002, respectively). Patients in C-ACB and PCA + sACB groups had less breakthrough pain (NRS > 5) than the IV-PCA group at POD1 (*p* = 0.007). On POD2, C-ACB was statistically superior to IV-PCA (*p* = 0.011) in terms of breakthrough pain. Postoperative pain NRS was lower in the C-ACB and PCA + sACB groups than in the IV-PCA group (*p* = 0.025 and *p* = 0.019, respectively). The total number of anti-emetics consumption was lower in C-ACB and PCA + sACB groups than in the IV-PCA group (*p* = 0.003 and *p* = 0.002, respectively).

**Conclusion:**

PCA + sACB not only reduced patients’ need for rescue opioids, but also decreased the number of breakthrough pain and anti-emetics compared to IV-PCA in early postoperative days after TKA. However, C-ACB and PCA + sACB did not differ significantly in analgesic efficacy or opioid-related side effects. PCA + sACB can be as effective as C-ACB for patients undergoing TKA.

**Level of evidence:**

Retrospective cohort study, level III.

## Introduction

Total knee arthroplasty (TKA) is regarded as an effective surgical treatment for severe degenerative osteoarthritic knees with excellent surgical outcomes [1]. As the population ages, there will be an increasing demand for joint replacement surgery [2]. Meanwhile, moderate to severe postoperative pain can impair recovery and rehabilitation after TKA. Adequate postoperative pain control not only reduces pain, but also decreases opioid consumption, consequently decreasing opioid-related adverse events [3–5]. However, effective pain control remains an issue due to its subjective nature and patient diversity [4]. Several postoperative analgesic regimens are utilized to maximize analgesic effects and minimize possible undesired adverse events [3]. In the past, epidural analgesia was regularly used as postoperative analgesic regimen after TKA. However, it had adverse effects such as motor nerve block, urinary retention, and hypotension, leading to limited use of epidural analgesia. Intravenous patient-controlled analgesia was developed and was used widely for pain management to give rapid, simple and appropriate pain control [2]. Opioids are delivered easily by the patient by pressing a button. Recently, peripheral nerve blockade are becoming widely accepted due to reduction of opioid consumption while having adequate pain relief [6]. Adductor canal block (ACB) is among them and has become popular because patients have better postoperative rehabilitation and opioid-related adverse effects. It can be administered continuously using a catheter, or given as a single shot [7]. Clinical results between continuous adductor canal block (C-ACB) and single injection are debatable [8]. Recent studies have reported that peripheral nerve block has better analgesic effects and fewer opioid-related adverse effects than IV-PCA [9, 10]. Other studies have reported that ACB has only a comparable pain control effect over IV-PCA [11, 12]. However, studies evaluating IV-PCA combined with single shot ACB (PCA + sACB) in terms of analgesic efficacy and opioid-related side effects are lacking. As the majority of patients experience block resolution and breakthrough pain early after surgery, long-lasting analgesic regimens are warranted [13].

The objective of this study was to compare three analgesic methods (IV-PCA, C-ACB, PCA + sACB) for patients undergoing TKA. We hypothesized that patients with C-ACB or PCA + sACB would need less rescue opioids and experience less breakthrough pain than patients with IV-PCA. In addition, we hypothesized that C-ACB and PCA + sACB groups would show similar analgesic effects and opioid-related side effects. The primary outcome was rescue opioids consumption. Another objective of this study was to evaluate the expected number of breakthrough pain, pain numerical rating scale (NRS), and anti-emetics administration.

## Materials and methods

**Fig. 1:**
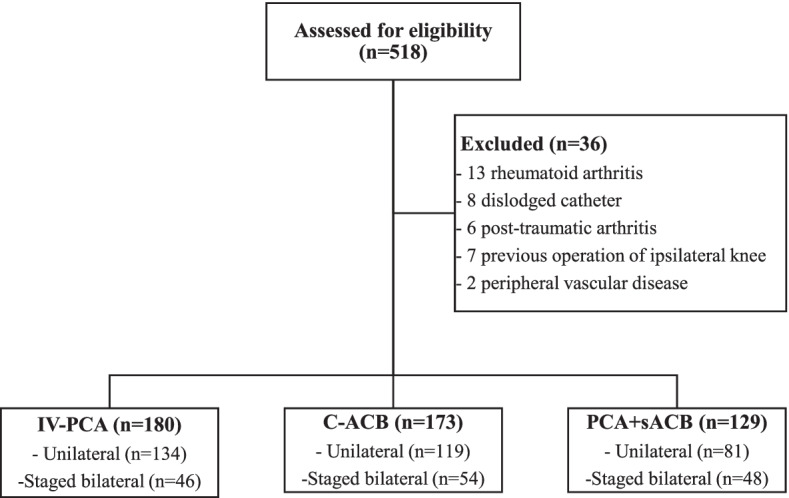
Flow chart of included patients. Postoperative pain control methods were divided into three groups: IV-PCA (*n* = 180), C-ACB (*n* = 173) and PCA + sACB (*n* = 129)

This study was approved by the Institutional Review Board (IRB) of Seoul National University Hospital (IRB No. 2203–086-1308). Informed consent was waived due to the retrospective nature of this study. Patients with end-stage osteoarthritis undergoing primary TKA (unilateral or staged bilateral) from September 2019 to September 2020 were included in our study. Exclusion criteria were: patients with rheumatoid arthritis, post-traumatic arthritis, previous operative history of the ipsilateral knee, allergy to local anesthetics, peripheral vascular disease, contraindication to nerve block (localized infection, preexisting neurological disorder), history of chronic pain requiring long-acting opioid use, and patients who had dislodged catheter of IV-PCA or C-ACB. Medical records of patients who underwent primary TKA were retrieved from our institute database. A total of 518 patients were identified as having primary TKA. Of them, 482 patients met our inclusion and exclusion criteria. Postoperative pain control methods were divided into three groups: IV-PCA (*n* = 180), C-ACB (*n* = 173) and PCA + sACB (*n* = 129) (Fig. 1). The decision to administer which of the three analgesic regimens was made by both the anesthesiologist and the orthopedic surgeon according to their preference at the time of surgery. Patient charts were reviewed to evaluate demographics and characteristics such as age, body mass index (BMI), and sex (Table 1). Pain NRS and numbers of opioids and anti-emetics consumption were evaluated from postoperative day (POD) 1 to POD 5. Measurements at the day of surgery (POD0) were not included because the effect of spinal anesthesia needed several hours to subside. In addition, single shot ACB was given at POD1. Pain-NRS was measured three times a day (0AM, 8AM, 4PM, a total of 15 times). Pain-NRS score of more than five points was defined as breakthrough pain. Their frequency was checked. The number of breakthrough pain per patient was calculated throughout the hospital stay. Nursing and physician charts were examined to document any dislodged catheter.

**Table 1:** Demographic characteristics of patients included in this study

	IV-PCA (*n *= 180)	C-ACB (*n *= 173)	PCA + sACB (*n* = 129)	*p*-value
Age (years)	72.6 ± 5.5	71.6 ± 5.1	72.0 ± 5.9	*p* = 0.235^a^
BMI (kg/m^2^)	26.8 ± 4.1	27.3 ± 4.0	26.3 ± 2.8	*p* = 0.067^a^
Sex				
Male (%)	13	16	19	*p* = 0.374^b^
Female (%)	87	84	81	

^†^Values are presented as mean ± standard deviation

^‡^*IV-PCA* Intravenous patient-controlled analgesia, *C-ACB* Continuous adductor canal block, *sACB* Single-shot adductor canal block, *BMI* Body mass index

^a^ One-way analysis of variance

^b^ Pearson’s chi-squared test

All patients received a standardized, multimodal analgesic. Preoperative celecoxib (400 mg), pregabalin (75 mg), and tramadol/acetaminophen (37.5 mg/325 mg) were given on the night before surgery. Dexamethasone (10 mg) was administered intravenously at 1 h before surgery. Spinal anesthesia was performed by an anesthesiologist, with bupivacaine (12 mg) as the intrathecal agent. Intraoperative periarticular injections (ropivacaine 9 mg + ketorolac 30 mg) were injected by the orthopedic surgeon. IV-PCA (nefopam 80 mg + Fentanyl 1000 mcg) was administered on either forearm right after surgery at a basal infusion rate of 15 mcg/ml/hr and a bolus of 15 mcg/mL with a lockout interval of 15 min. C-ACB (68 ml of ropivacaine at 7.5 mg/ml + 182 ml of normal saline, total 250 ml) was given after surgery by an anesthesiologist in the operating room. When administrating C-ACB, standard skin preparation and sterile draping were applied around the proximal femoral crease. A 21-gauge spinal needle was advanced near the femoral nerve sheath under ultrasound and nerve stimulator guidance. After confirming no motor response of quadriceps muscle (less than 0.45 mA), the catheter was advanced and attached to the skin at an infusion rate of 5 ml/hr. In the PCA + sACB group, IV-PCA was administered as described above. sACB (20 cc of ropivacaine at 7.5 mg/ml + 5 cc of 2% lidocaine) was performed 24 h after surgery by an orthopedic specialist who participated in the surgery. Standard skin preparation and sterile draping were applied around the distal femoral crease. Under ultrasound guidance, a 21-gauge spinal needle was advanced into the adductor canal at mid-thigh level where a bolus of 20 cc was injected.

Postoperative analgesia was standardized among the three groups: dexamethasone (10 mg) at 9 AM on POD 1 and celecoxib (200 mg q12 hr) during hospital stay. The number of rescue opioid analgesics given additionally at patients’ request from POD 1 to POD 5 (morphine 5 mg or oxycodone 5 mg) were counted. To prevent postoperative nausea and vomiting, standardized anti-emetics were given preoperatively and postoperatively. Palonosetron (0.075 mg/1.5 mL) was given 1 h before surgery and ramosetron (0.3 mg/2 mL) was given daily from POD 1 to POD 3. Additional anti-emetics (ramosetron 0.3 mg/2 mL and/or granisetron 1 mg/1 mL and/or metoclopramide 10 mg/2 mL) were given at the patients’ request.

### Statistical analysis

The sample size was based on a pilot study involving 20 patients per group not enrolled in the main study. A minimum of 44 individuals were required for each group to achieve a 30% difference in postoperative opioid consumption, with a two-sided alpha level of 0.05 and a power of 90%. All statistical analyses were performed using IBM SPSS Statistics 25 (IBM, Chicago, IL, USA). For categorical data, Pearson’s chi-squared test were used. For continuous data, normality was analyzed and one-way analysis of variance (ANOVA) was performed. Post hoc analysis (Bonferroni’s method) was used to adjust for multiple comparisons among groups. A *p*-value < 0.05 was considered statistically significant.

## Results

The three groups were similar in age (IV-PCA = 72.6 years, C-ACB = 71.6 years, PCA + sACB = 72.0 years, *p* = 0.235), BMI (IV-PCA = 26.8, C-ACB = 27.3, PCA + sACB = 26.3, *p* = 0.067), and sex (males: IV-PCA = 13%, C-ACB = 16%, PCA + sACB = 18%, *p* = 0.374). A review of medical records showed 8 dislodged catheters, which were exclusively in the C-ACB group.

### Primary outcome

**Fig. 2:**
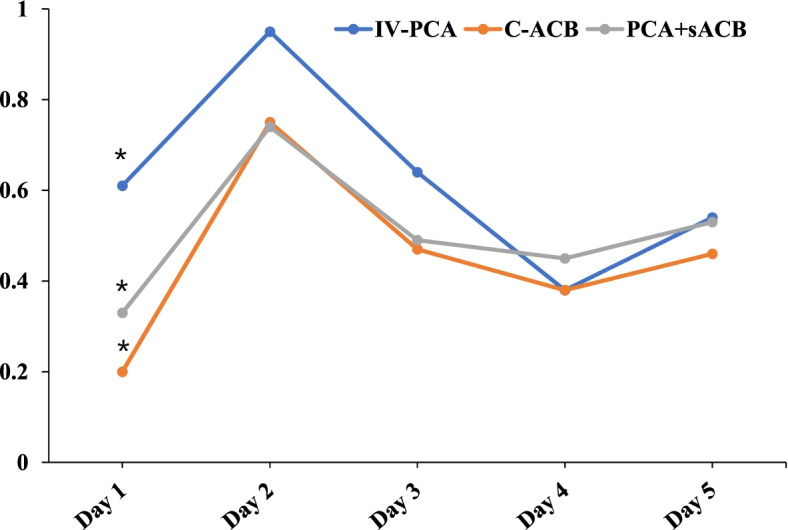
Rescue opioid consumption from POD1 to POD5. The number of rescue opioid usage was lower in C-ACB and PCA + sACB groups than in the IV-PCA group, especially on POD1 (*p* < 0.001). There was no difference in rescue opioid consumption between C-ACB and PCA + sACB groups

Regarding rescue opioid consumption, PCA + sACB and C-ACB was statistically superior to IV-PCA at POD1 (*p* < 0.001) and graphically superior from POD2 to POD3. After that, there was not a significant difference between the three analgesic options. There was no difference in rescue opioid consumption between C-ACB and PCA + sACB groups. (Fig. 2, Table 2).

**Table 2:** Number of rescue opioid usage from POD1 to POD5

No. of opioid usage	POD1	POD2	POD3	POD4	POD5
IV-PCA (*n* = 180)	0.61 ± 0.88	0.95 ± 1.30	0.64 ± 1.05	0.38 ± 0.74	0.54 ± 0.97
C-ACB (*n* = 173)	0.20 ± 0.46	0.75 ± 0.86	0.47 ± 0.71	0.38 ± 0.60	0.46 ± 0.69
PCA + sACB (*n* = 129)	0.33 ± 0.66	0.74 ± 1.02	0.49 ± 0.77	0.45 ± 0.81	0.53 ± 0.82
*p*-value	< 0.001 ^a^	0.143 ^a^	0.121 ^a^	0.612 ^a^	0.633 ^a^
IV-PCA vs. C-ACB	* < 0.001 ^b^				
IV-PCA vs. PCA + sACB	*0.002 ^b^				
C-ACB vs. PCA + sACB	0.384 ^b^				

^†^Values are presented as mean ± standard deviation

^‡^*IV-PCA* Intravenous patient-controlled analgesia, *C-ACB* Continuous adductor canal block, *sACB* Single-shot adductor canal block, *POD* Postoperative day

^a^ One-way analysis of variance

^b^ Post hoc (Bonferroni’s method) analysis

^*^ Statistically significant at *p* < 0.05

### Secondary outcomes

**Fig. 3:**
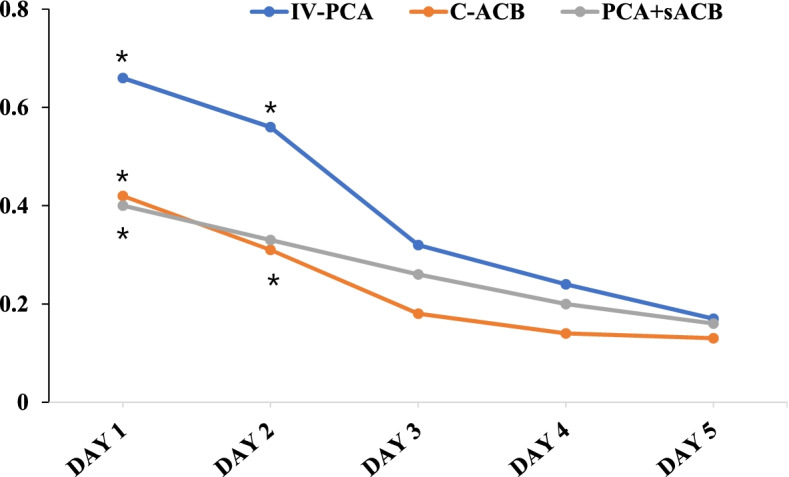
At POD1, the expected value of breakthrough pain was lower in C-ACB and PCA + sACB groups than in the IV-PCA group (*p* = 0.007). At POD2, C-ACB was statistically superior to IV-PCA (*p* = 0.011) and PCA + sACB was graphically superior to IV-PCA. Afterwards, C-ACB and PCA + ACB was graphically better than IV-PCA throughout the admission period

Frequencies of breakthrough pain from POD1 to POD5 were 12.9%, 7.9%, and 9.0% in IV-PCA, C-ACB, and PCA + sACB group, respectively (*p* < 0.001) (Table 3). Expected values of breakthrough pain per patient from POD1 to POD5 are shown in Table 4 and Fig. 3. At POD1, the expected value of breakthrough pain was lower in C-ACB and PCA + sACB groups than in the IV-PCA group (*p* = 0.007). At POD2, C-ACB was statistically superior to IV-PCA (*p* = 0.011) and PCA + sACB was graphically superior to IV-PCA. Afterwards, C-ACB and PCA + ACB was graphically better than IV-PCA throughout the admission period. In terms of pain NRS throughout hospital stay, IV-PCA group experienced more pain than the other groups at POD1 (*p* = 0.025 at 8AM and *p* = 0.019 at 4PM). Afterwards, there were no pain NRS difference between the three groups. (Table 5). The total number of additional anti-emetics usage per patient was lower in C-ACB and PCA + sACB groups than in the IV-PCA group (*p* = 0.003 and *p* = 0.002, respectively). Anti-emetics usage between C-ACB and PCA + sACB groups showed similar results (*p* = 1.000) (Table 6).

**Table 3:** Total frequencies of breakthrough pain from POD1 to POD5

Pain NRS	IV-PCA (*n *= 180)	C-ACB (*n *= 173)	PCA + sACB (*n *= 129)	*p*-value ^a^
breakthrough	12.9%	7.9%	9.0%	*0.01
mild	87.1%	92.1%	91.0%	

^†^*NRS* Numerical rating scale, *IV-PCA* Intravenous patient-controlled analgesia, *C-ACB* Continuous adductor canal block, *sACB* Single-shot adductor canal block, *POD* Postoperative day

^a^ Pearson’s chi-squared test

^*^ Statistically significant at *p* < 0.05

**Table 4:** Expected value of breakthrough pain per patient from POD1 to POD5

	IV-PCA (*n* = 180)	C-ACB (*n* = 173)	PCA + sACB (*n* = 129)	*p*-value ^a^
POD1	0.66	0.42	0.40	0.007 ^b^
POD2	0.56	0.31	0.33	0.011 ^c^
POD3	0.32	0.18	0.26	0.111
POD4	0.24	0.14	0.20	0.338
POD5	0.17	0.13	0.16	0.658

^†^*IV-PCA* Intravenous patient-controlled analgesia, *C-ACB* Continuous adductor canal block, *sACB* Single-shot adductor canal block, *POD* postoperative day

^a^ One-way analysis of variance

^b^ Post hoc (Bonferroni’s method) analysis showed that expected value of C-ACB or PCA + sACB was lower than that of IV-PCA

^c^ Post hoc (Bonferroni’s method) analysis showed that expected value of C-ACB was lower than that of IV-PCA

^*^ Statistically significant at *p* < 0.05

**Table 5:** Pain NRS of IV-PCA, C-ACB, and PCA + sACB

Pain-NRS	IV-PCA (*n* = 180)	C-ACB (*n* = 173)	PCA + sACB (*n* = 129)	*p-*value^a^
POD1				
0A	3.52 ± 1.91	3.41 ± 1.77	3.49 ± 1.63	0.836
8A	4.57 ± 2.21	4.05 ± 1.75	4.11 ± 1.80	*0.025 ^b^
4P	4.39 ± 1.86	3.99 ± 1.53	3.91 ± 1.55	*0.019 ^c^
POD2				
0A	4.18 ± 1.78	3.88 ± 1.38	3.83 ± 1.39	0.091
8A	3.84 ± 1.80	3.65 ± 1.40	3.85 ± 1.54	0.451
4P	3.69 ± 1.66	3.73 ± 1.36	3.81 ± 1.41	0.781
POD3				
0A	3.70 ± 1.68	3.68 ± 1.42	3.73 ± 1.44	0.958
8A	3.63 ± 1.75	3.60 ± 1.48	3.62 ± 1.45	0.980
4P	3.38 ± 1.37	3.19 ± 1.08	3.47 ± 1.32	0.131
POD4				
0A	3.49 ± 1.34	3.18 ± 1.08	3.40 ± 1.16	0.056
8A	3.74 ± 1.63	3.49 ± 1.43	3.57 ± 1.42	0.274
4P	3.53 ± 1.32	3.53 ± 1.25	3.61 ± 1.18	0.815
POD5				
0A	3.29 ± 1.23	3.42 ± 1.26	3.59 ± 1.18	0.115
8A	3.29 ± 1.42	3.57 ± 1.35	3.50 ± 1.30	0.154
4P	3.44 ± 1.37	3.47 ± 1.12	3.52 ± 1.20	0.855

^†^Values are presented as mean ± standard deviation

^‡^*NRS* Numerical rating scale, *IV-PCA* Intravenous patient-controlled analgesia, *C-ACB* Continuous adductor canal block, *sACB* Single-shot adductor canal block, *POD* Postoperative day

^a^ One-way analysis of variance

^b^ Post hoc (Bonferroni’s method) analysis showed that Pain NRS of C-ACB was lower than that of IV-PCA

^c^ Post hoc (Bonferroni’s method) analysis showed that Pain NRS of PCA + sACB was lower than that of IV-PCA

^*^ Statistically significant at *p* < 0.05

**Table 6:** Total number of additional anti-emetics usage from POD1 to POD5

	No. of anti-emetics usage
IV-PCA (*n *= 180)	4.54 ± 1.14
C-ACB (*n* = 173)	4.23 ± 0.73
PCA + sACB (*n* = 129)	4.19 ± 0.61
*p*-value	0.001 ^a^
IV-PCA vs. C-ACB	*0.003 ^b^
IV-PCA vs. PCA + sACB	*0.002 ^b^
C-ACB vs. PCA + sACB	1.000 ^b^

^†^Values are presented as mean ± standard deviation

^‡^*IV-PCA* Intravenous patient-controlled analgesia, *C-ACB* Continuous adductor canal block, *sACB* Single-shot adductor canal block, *POD* Postoperative day

^a^ One-way analysis of variance

^b^ Post-hoc (Bonferroni’s method) analysis

^*^ Statistically significant at *p* < 0.05

## Discussion

In this retrospective cohort study, IV-PCA, C-ACB, and PCA + sACB were compared in terms of opioid consumption, breakthrough pain, pain NRS, and anti-emetics consumption after TKA. In literature, IV-PCA alone after TKA might require considerable opioids, with epidural analgesia showing greater risk of adverse effects such as urinary retention or hypotension [14, 15]. As sACB shows effectiveness on pain and ambulation after TKA but may not last over 24 h, [6, 16] IV-PCA was combined with sACB postoperatively. sACB was injected at 24 h after surgery because the effect of spinal anesthesia and periarticular injection might last until the day of surgery [17]. As a result, PCA + sACB was superior in managing breakthrough pain than IV-PCA at POD1 in this study. Consequently, rescue opioid required to relieve severe pain was less in the PCA + sACB group than in the IV-PCA group.

Whether C-ACB is better than sACB in terms of analgesia and opioid-related adverse effects is controversial [18–20]. Our findings in this study showed that PCA + sACB was similar to C-ACB in primary and secondary outcomes. Some studies have suggested that C-ACB may cause a decrease in quadriceps strength and increase the risk of falling [18, 21, 22]. In addition, placement of the catheter at the operated leg can impair postoperative ambulation, and early dislodgement may cause uncontrolled pain [23]. In our study, 8 catheters were dislodged in the C-ACB group while the other groups did not show any dislodgement. Although the number is low, C-ACB may be in more risk of dislodgement than the other analgesic methods. Meanwhile, sACB can be performed without the concern of the catheter being dislodged while postoperative rehabilitation.

This study has several limitations. First, our study design was retrospective. It might have potential bias inherent to retrospective studies. Further prospective studies are needed to compare the three pain control methods. Second, this study had a relatively small sample size, which might have affected our study results. Studies with larger sample sizes are warranted to compare efficacies of IV-PCA, C-ACB, and PCA + sACB. Third, pain-NRS scores were counted three times a day (0A, 8A, 4P) for a total of 15 times (from POD1 to POD5), which could not reflect the continuous fluctuation of pain throughout patient’s hospital stays. Fourth, patients were given standardized, routine anti-emetics until POD3 because removal of these agents would not be ethical. Accordingly, the efficacy of controlling postoperative nausea and vomiting might not be accurate. Fifth, analgesic provider variation, difference in skill, and patient nociception variability might have affected results of this study. Sixth, the study groups included more than 80% female. This is because the study was performed in South Korea, in which osteoarthritis is more prevalent in females. Data from the Health Insurance Review and Assessment Service (HIRA) of South Korea showed that from 2001 through 2010, a total of 390,888 primary TKAs were performed and 90% of procedures were performed on females throughout the study period [24]. Difference in ethnicity may cause bias in our study results.

## Conclusion

C-ACB and PCA + sACB reduced the use of rescue opioids, frequency of breakthrough pain, and anti-emetics consumption compared to IV-PCA after TKA. In terms of analgesic effects and opioid-related side effects, C-ACB and PCA + sACB showed similar results.

## Abbreviations

TKA: Total knee arthroplasty
IV-PCA: Intravenous patient-controlled analgesia
C-ACB: Continuous adductor canal block
PCA + sACB: Intravenous patient-controlled analgesia combined with single shot adductor canal block
POD: Postoperative day
NRS: Numerical rating scale
BMI: Body mass index
